# Global Research Trends in Gastric Cancer Epigenetics From 2005 to 2025

**DOI:** 10.7759/cureus.106195

**Published:** 2026-03-31

**Authors:** Didi Yuan, Yanling Li, Hong Yi, Yan Lei, Yanhong Zhou

**Affiliations:** 1 Department of Blood Transfusion, The Affiliated Cancer Hospital of Xiangya School of Medicine Central South University, Hunan Cancer Hospital, Changsha, CHN; 2 Basic School of Medicine, Central South University, Central South University, Changsha, CHN; 3 Department of Nuclear Medicine, The Affiliated Cancer Hospital of Xiangya School of Medicine Central South University, Hunan Cancer Hospital, Changsha, CHN; 4 Research Center of Carcinogenesis and Targeted Therapy, Xiangya Hospital of Central South University, Changsha, CHN; 5 Cancer Research Institute, Basic School of Medicine, Central South University, Changsha, CHN

**Keywords:** bibliometrics, citespace, epigenetic, gastric cancer, vosviewer

## Abstract

Background: Gastric cancer (GC) is one of the most common malignant tumors in the digestive tract, characterized by a high degree of malignancy and a poor prognosis. Complex disease heterogeneity, late diagnosis, and suboptimal treatment often lead to poor prognoses for patients. In addition to genetic alterations and environmental factors, changes in epigenetic mechanisms also play an important role in the occurrence and progression of GC. Revealing the current research status of epigenetics in GC has important guiding significance for the diagnosis and treatment of GC, as well as for future research directions.

Methodology: In this study, we used specific keywords to search for publications related to gastric cancer epigenetics (GCE) published in the past 20 years in the Web of Science Core Collection database. The VOSviewer software (Version 1.6.20; Center for Science and Technology Studies (CWTS), Leiden University, Leiden, The Netherlands) was used to conduct clustering and co-occurrence analysis on countries, authors, institutions, and keywords in the publications. CiteSpace software (Version 6.3.1R6; Chaomei Chen, College of Computing and Informatics, Drexel University, Philadelphia, PA) was used for burst analysis and timeline clustering analysis.

Result: A total of 4,655 papers on GC in the field of epigenetics were retrieved. China is the country with the largest number of published papers (*n* = 1,876), while the United States has the highest centrality (0.32). Nanjing University ranks first among all institutions in terms of the number of published papers (*n* = 175). The author Ushijima Toshikazu from Japan has the highest number of published papers (*n* = 48). Yuan Yuan is a representative author of the recent research hotspots. In the keyword ranking, keywords related to DNA methylation are relatively numerous. Tumor microenvironment and immune infiltration are key buzzwords with research potential.

Conclusions: Based on bibliometric methods, this study provides new insights into the research progress of GC from the perspective of epigenetics and offers new ideas and inspirations for future GC research.

## Introduction

According to data from the World Health Organization (WHO) and the International Agency for Research on Cancer (IARC), gastric cancer (GC) ranks fourth in global cancer incidence and second in cancer-related mortality. Annually, over one million new cases are diagnosed, resulting in approximately 780,000 deaths worldwide. Alarmingly, the mortality rate from GC continues to rise, and the age of onset is trending younger [1]. Most patients are diagnosed at intermediate or advanced stages, often with distant metastasis, leading to a dismal 5-year survival rate of only about 20%. Compounding this challenge, the early symptoms of GC lack specificity, resulting in a stage I detection rate of merely 10% [2]. While GC pathogenesis involves a complex interplay of genetic and environmental factors, such as smoking, alcohol consumption, and Helicobacter pylori infection, studies indicate that environmental factors are the primary drivers of disease onset [3].

Epigenetics serves as a critical bridge connecting these environmental exposures to disease phenotypes. First conceptualized by Conrad Waddington in 1942 as *epigenetic inheritance*, the field has evolved to define heritable changes in gene expression that occur without alterations in the underlying DNA nucleotide sequence [4]. Key epigenetic mechanisms currently identified include gene imprinting, chromatin remodeling, DNA methylation, histone modification, and non-coding RNA regulation. These modifications play pivotal roles in tumor initiation and progression by dynamically influencing the tumor microenvironment and regulating oncogene or tumor suppressor activity [5]. Despite rapid advancements, the sheer volume of literature on GC epigenetics makes it challenging for researchers to synthesize existing knowledge, identify emerging trends, and pinpoint critical gaps.

Bibliometrics offers a robust solution to this challenge by investigating the intrinsic characteristics and underlying patterns of scientific literature. By employing quantitative methods rooted in mathematics and statistics, bibliometrics analyzes the distribution structures, collaborative networks, and evolutionary trajectories of research outputs. Its application in the medical field enables researchers to visualize vast publication landscapes at both macroscopic and microscopic levels, thereby facilitating a rapid and comprehensive understanding of developmental trends within specific domains [6].

Therefore, there is an urgent need to comprehensively evaluate the global research landscape of gastric cancer epigenetics (GCE) to clarify its intellectual structure and future directions. The primary objective of this study is to systematically map the evolutionary trajectory and hotspots of GCE research over the past two decades. Specifically, by analyzing publication patterns and keyword dynamics across multiple dimensions, including contributing countries, institutions, and thematic clusters, this study aims to pinpoint under-explored epigenetic mechanisms and detect frontier research topics. Unlike traditional narrative reviews, our bibliometric approach provides a macroscopic view that explicitly guides future research priorities, helping scientists to optimize resource allocation toward promising diagnostic biomarkers and therapeutic targets, rather than merely summarizing past literature.

## Materials and methods

Data sources and retrieval strategies

The data for this study were retrieved from the Web of Science Core Collection (WoSCC) database, which is widely recognized as a reliable source for bibliometric analysis due to its comprehensive citation indexing and standardized data structure. Although multiple databases such as PubMed, Scopus, and Embase are commonly used in bibliometric studies, WoSCC was selected as the sole data source in this study because of its high-quality citation data and strong compatibility with bibliometric tools such as CiteSpace (Version 6.3.1R6; Chaomei Chen, College of Computing and Informatics, Drexel University, Philadelphia, PA) and VOSviewer (Version 1.6.20; Center for Science and Technology Studies (CWTS), Leiden University, Leiden, The Netherlands). In addition, integrating data from multiple databases may introduce inconsistencies in citation formats and duplicate records, potentially affecting the accuracy of network analysis. Therefore, to ensure data homogeneity and analytical robustness, only WoSCC was used in this study.

The retrieval period was set from January 1, 2005, to April 19, 2025. To ensure the comprehensiveness and accuracy of the search results, a combination of subject terms and free-text terms related to GC and epigenetics was used. The detailed search strategy is as follows: (TS=(gastric cancer) AND TS=(epigenetic OR DNA methylation OR chromatin remodeling OR genomic imprinting OR histone modification OR RNA modification OR RNA methylation OR m6A)). The inclusion criteria were as follows: (1) publications related to GC and epigenetics; (2) articles and reviews; and (3) publications written in English. The exclusion criteria included: (1) meeting abstracts, editorial materials, letters, and other non-research articles; and (2) duplicate records. All retrieved records were downloaded in plain text format, including full records and cited references, for subsequent bibliometric analysis.

Data collection and screening process

**Figure 1:**
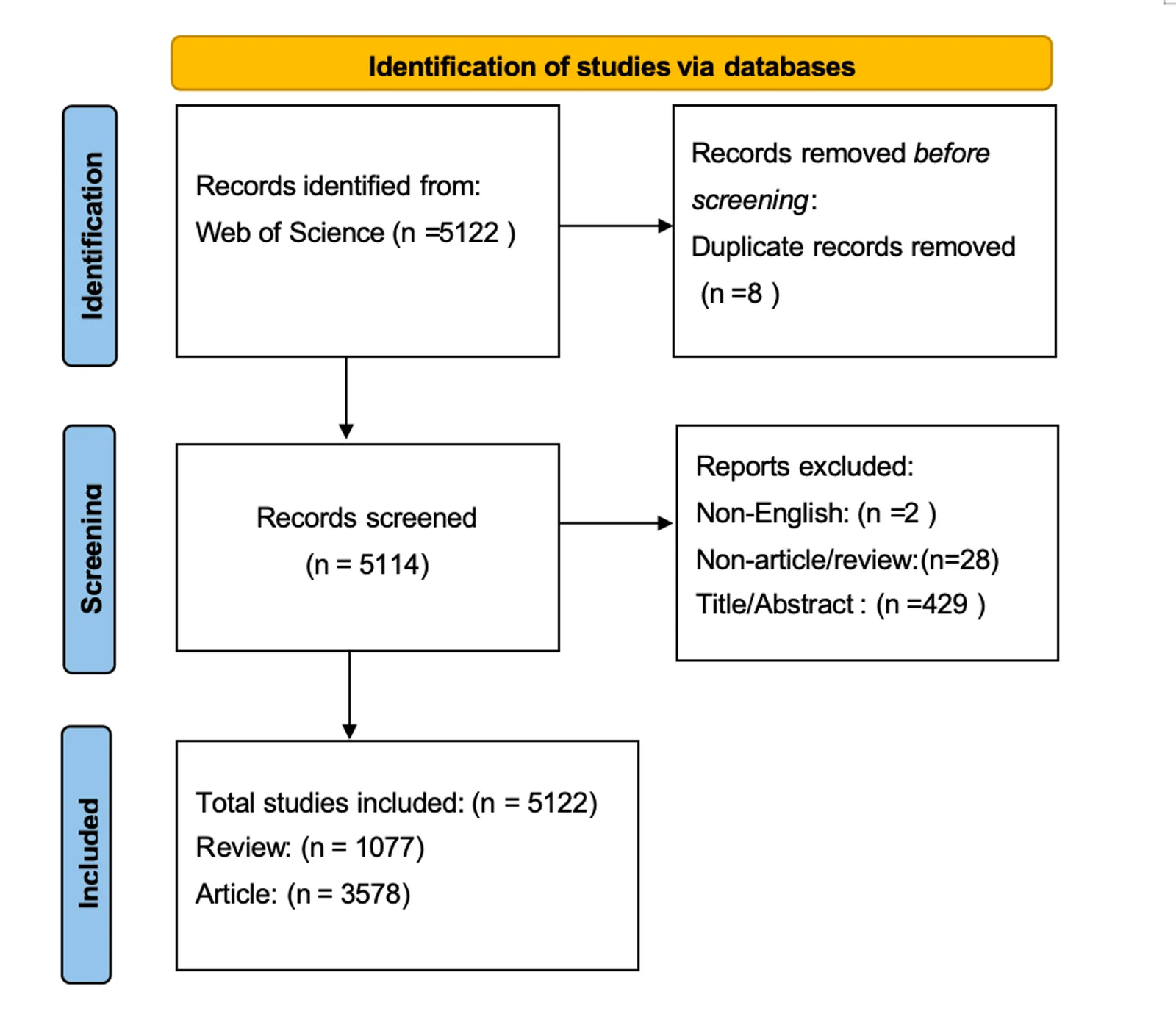
PRISMA 2020 flow diagram of the literature selection process. PRISMA, Preferred Reporting Items for Systematic Reviews and Meta-Analyses

The study selection process was conducted in accordance with the Preferred Reporting Items for Systematic Reviews and Meta-Analyses (PRISMA) 2020 guidelines. A total of 5,122 records were initially identified from the WoSCC. After removing 8 duplicate records, 5,114 records remained for screening. Following title and abstract screening, 429 records were excluded. A total of 4,685 full-text articles were assessed for eligibility. Among these, 30 records were excluded, including 2 non-English publications and 28 non-article/review types. Ultimately, 4,655 publications were included in the final bibliometric analysis, comprising 3,578 articles and 1,077 reviews. The detailed selection process is illustrated in Figure 1.

Data collection and analysis

The retrieved data were imported into CiteSpace (Version 6.3.1R6), VOSviewer (Version 1.6.20), and Scimago Graphica (Version 1.0.50; SCImago Research Group, Granada, Spain) for bibliometric analysis and visualization. To ensure the reliability and reproducibility of the results, all analyses were conducted using standardized procedures commonly applied in bibliometric studies.

Duplicate records were identified and removed using CiteSpace's built-in deduplication function, followed by manual verification to ensure data accuracy. The screening process was carefully conducted and cross-checked to maintain consistency. CiteSpace was primarily used to analyze collaboration networks among countries, institutions, and authors, as well as to detect keyword bursts and identify emerging research trends. VOSviewer was applied to construct and visualize co-authorship, co-citation, and keyword co-occurrence networks. Scimago Graphica was used to generate geographical distribution maps. The parameters in CiteSpace and VOSviewer were set based on commonly used standards in bibliometric studies. Specifically, threshold values such as the minimum number of publications or occurrences were adjusted according to the characteristics of the dataset to ensure meaningful visualization results. Unless otherwise specified, default settings were applied.

## Results

To address the objectives of this study, we systematically analyzed publication trends, countries, institutions, authors, journals, and keywords.

Time trend analysis of publication volume

**Figure 2:**
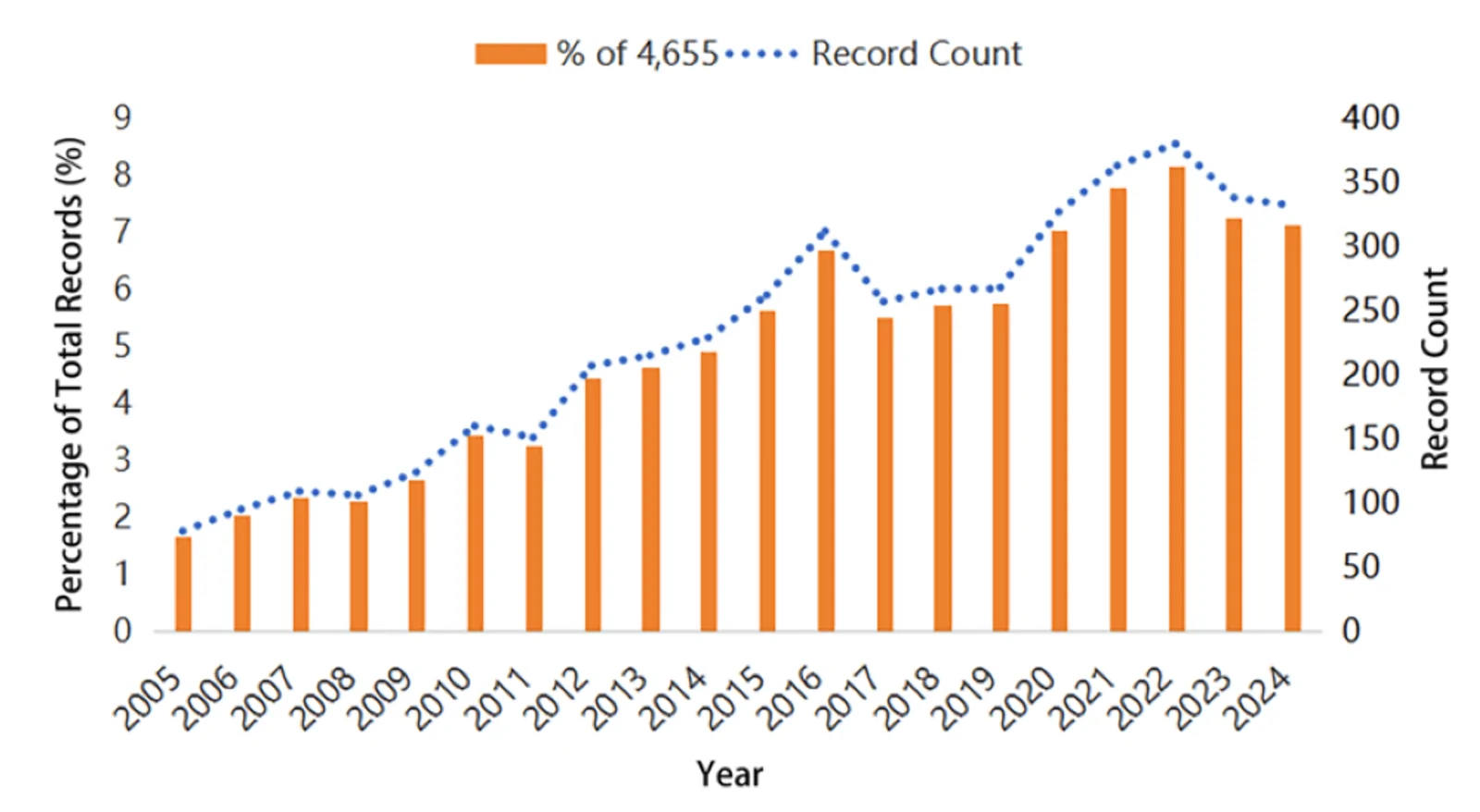
Analysis of the annual publication quantity of literature on GCE. GCE, gastric cancer epigenetics

To understand the overall trend in the publication of articles on GC in the field of epigenetics, we conducted a statistical analysis of the publication dates of 4,655 publications (Figure 2). To ensure that all retrieved literature aligns with the research content, we refined the search keywords. This refinement may have resulted in a small number of relevant publications not being retrieved. To more clearly present the trend in article publication, we excluded the article statistics for 2025. The statistical results show that since 2005, the number of published articles has increased annually, reaching peaks in 2016 (*n* = 312) and 2022 (*n* = 380). The emergence of each peak in the field of GCE may indicate greater investment in research funds, technological advancements, and an increase in academic productivity, etc. Currently, research in GCE has entered a period of stable development, suggesting that the field has achieved phased results. At this stage, summarizing and analyzing the current research using bibliometric methods can provide insights for predicting future research hotspots and directions.

Country

**Figure 3:**
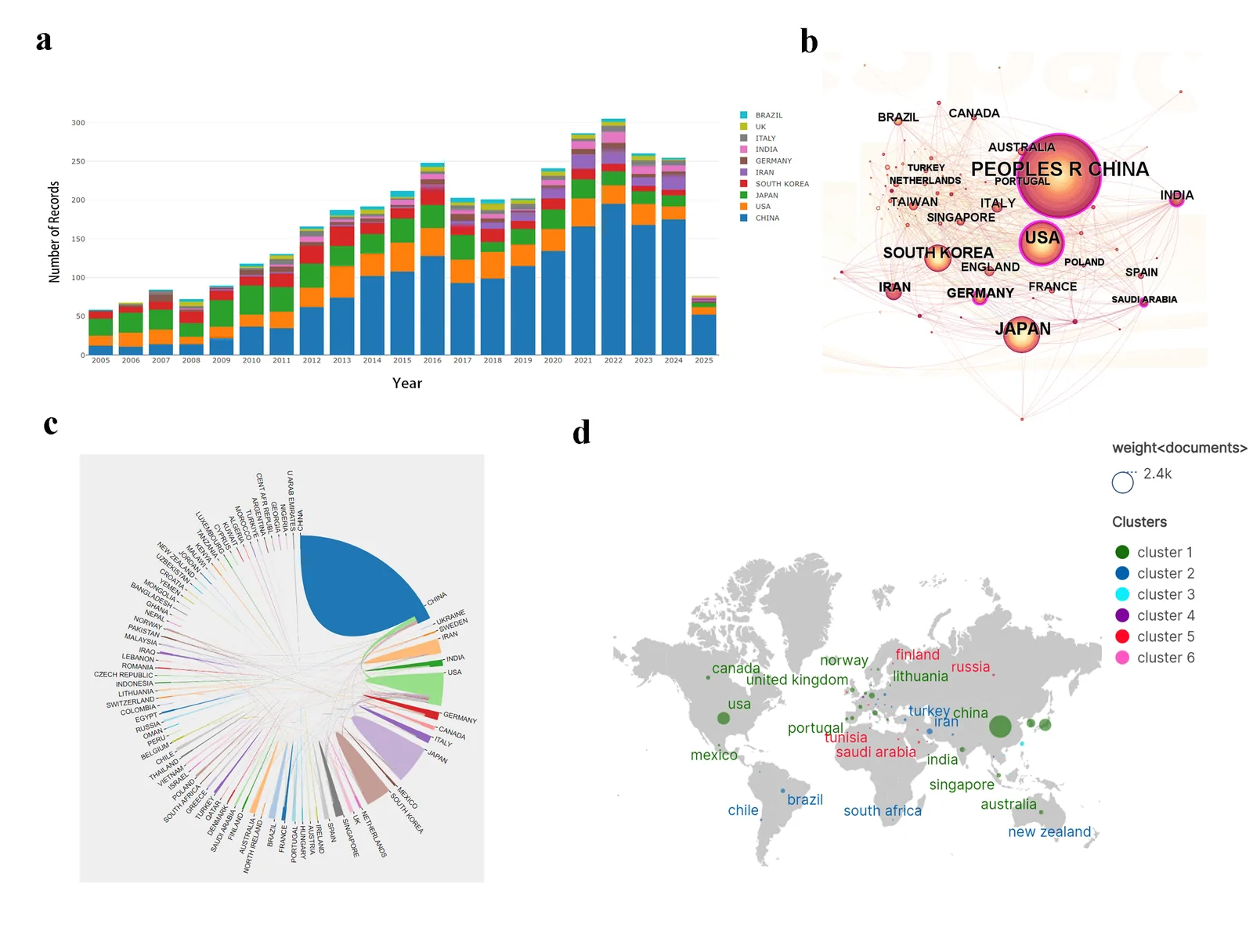
Visual analysis of gastric cancer epigenetics (GCE) in countries. (a) Chart of the proportion of articles published by each country each year from 2005 to 2025; (b) National Centrality Network diagram; (c) National Cooperation Network Map; (d) National Cooperation Clustering Map. Note: (a) The figure is drawn using a bibliometric website. The color represents the country, and the height represents the number of documents. (b) The figure was drawn using CiteSpace software. The size of the nodes represents the publication volume of the literature, and the thickness of the purple outer circle represents centrality. (c) The figure is drawn using a bibliometric website, with colors representing countries and lines representing cooperation routes. (d) The graph was drawn using Scimago Graphica software. The colors represent clustering, and the node size represents the number of documents.

According to the statistics, the 4,655 documents primarily originate from 88 countries. Among these, the top three countries with the highest number of publications are China (*n* = 1,876, 40.30%), the United States (*n *= 437, 9.39%), and Japan (*n* = 301, 6.47%) (Table 1). The proportion of literature from China has reached 40.30%, which is more than four times that of the United States. This indicates that China may have a relatively high research productivity and substantial funding support in the GCE research field. Figure 3a illustrates the annual publication trends of literature from various countries. The results show that the annual publication volumes from the United States and Japan have stabilized, while the publication volume from China has continued to increase, reaching its peak in 2022. This not only suggests that Chinese researchers have a high level of enthusiasm for GCE research but may also imply that the incidence of GC in China is relatively high [7].

**Table 1:** The top 20 countries in terms of the number of published documents.

Rank	Country	Record count	% of 4,655	Centrality
1	China	1876	40.30	0.13
2	United States	437	9.39	0.32
3	Japan	301	6.47	0.08
4	South Korea	161	3.46	0.07
5	Iran	146	3.14	0.06
6	India	97	2.08	0.11
7	Germany	85	1.83	0.19
8	Italy	84	1.80	0.08
9	England	58	1.25	0.05
10	Canada	53	1.14	0.01
11	Australia	52	1.12	0.04
12	Brazil	52	1.12	0.01
13	France	49	1.05	0.10
14	Singapore	49	1.05	0.01
15	Spain	42	0.90	0.04
16	Saudi Arabia	31	0.67	0.16
17	Turkey	28	0.60	0.00
18	Poland	24	0.52	0.02
19	Mexico	23	0.49	0.00
20	Pakistan	22	0.47	0.07

Centrality is an indicator that assesses the importance of a node in a network by measuring the number of shortest paths passing through it. In this study, the three countries with the highest centrality values were the United States (0.32), Germany (0.19), and Saudi Arabia (0.16), followed by China (0.13), India (0.11), and France (0.10) (Table 1). Figure 3b visually presents the centrality of various countries. In this figure, the thickness of the purple outer circle of a node is positively correlated with its centrality, while the size of the node represents the country's contribution in terms of the number of publications. Although the United States does not have the highest number of published documents, it has the highest centrality, indicating that it holds a core position in international cooperation. The node for Saudi Arabia is relatively small, but its purple outer circle is thicker, indicating that despite having fewer publications, the country is highly involved in international cooperation.

In order to further understand the cooperation among countries, we constructed a national cooperation network diagram (Figure 3c). In this diagram, different colors are used to distinguish each country, and the lines represent the cooperation between countries. The cooperation network of the United States is extremely complex and involves numerous countries, further validating the central position of the United States in the research of GCE. Among China's cooperative connections, the connection with the United States is the most prominent, indicating that the United States is China's main cooperative partner in this field. Furthermore, based on the link strength among countries, we divided these countries into six clusters and distinguished them with different colors (Figure 3d). Among these clusters, the green cluster includes countries such as China, the United States, Canada, the United Kingdom, and Australia, forming the largest cluster. The red cluster is centered around France and Russia, while the blue cluster is centered around Brazil and Iran.

Institutions

Research indicates that a total of 3,733 institutions have participated in the research of GCE. Table 2 lists the top 20 research institutions in terms of publication volume. Among these, Nanjing University (*n* = 175), Shanghai Jiao Tong University (*n* = 146), and the National Cancer Center of Japan (*n* = 142) rank as the top three institutions. Of the top 20 institutions, 14 are from China, with the remaining institutions from the United States, Japan, Singapore, and South Korea. The half-life of an institution is typically used to measure the academic influence and sustainability of the articles it publishes. A longer half-life indicates that the institution's articles have been cited over a longer time span in the academic community, reflecting that its research findings hold high long-term academic value. In this study, the Chinese Academy of Sciences (7.5) and Zhengzhou University (6.5) had the highest half-lives, indicating that the articles published by these two institutions in the field of GCE have a relatively strong influence.

**Table 2:** The top 20 countries in terms of the number of published documents.

Rank	Affiliations	Record count	Country	Half life
1	Nanjing Medical University	175	China	4.5
2	Shanghai Jiao Tong University	146	China	5.5
3	National Cancer Center Japan	142	Japan	2.5
4	China Medical University	112	China	3.5
5	Zhengzhou University	108	China	6.5
6	Peking University	107	China	3.5
7	Sun Yat-sen University	107	China	5.5
8	The Chinese University of Hong Kong	105	China	0.5
9	Seoul National University	105	South Korea	2.5
10	The University of Tokyo	90	Japan	4.5
11	Fudan University	87	China	5.5
12	University of Texas System	79	The United States	2.5
13	National University of Singapore	74	Singapore	5.5
14	Johns Hopkins University	70	The United States	-0.5
15	Shandong University	70	China	4.5
16	Huazhong University of Science and Technology	66	China	4.5
17	Central South University	64	China	3.5
18	Southeast University	64	China	2.5
19	Chinese Academy of Sciences	60	China	7.5
20	Chinese Academy of Medical Sciences	59	China	3.5

**Figure 4:**
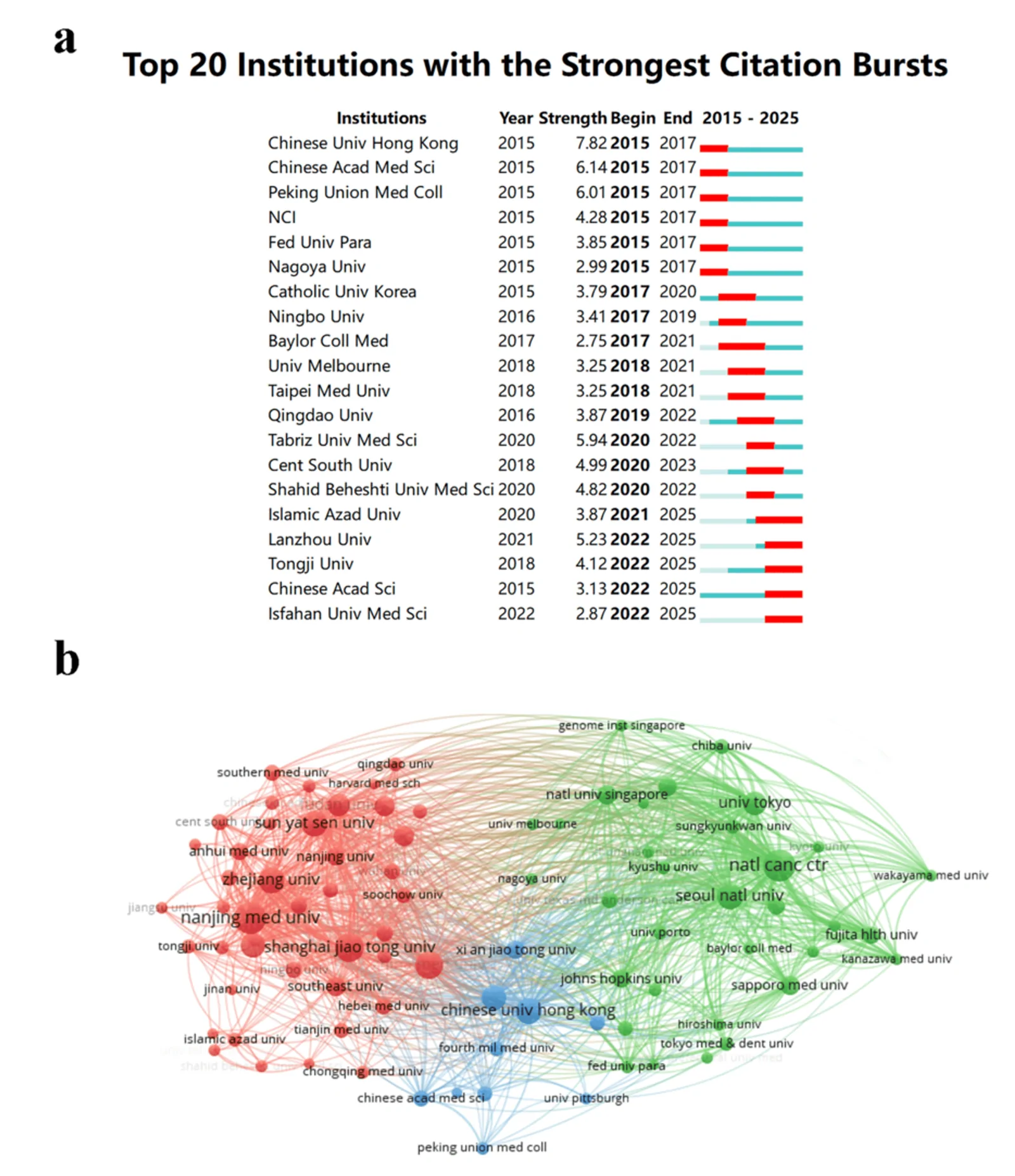
Visual analysis of research institutions in gastric cancer epigenetics (GCE). (a) Burst chart of the top 20 institutions; (b) institutional cooperation clustering network diagram. Note: (a) The figure was drawn using CiteSpace software. The red bands represent the burst period, while the blue bands represent the buffer period. (b) The figure was drawn using VOSviewer software. The colors represent clusters, the node size represents the number of documents, and the lines represent the link strength.

We analyzed the outbreak times of institutions (Figure 4a). The results indicate that the Chinese University of Hong Kong, the Chinese Academy of Medical Sciences, Peking Union Medical College, the National Cancer Center of Japan, Para Federal University, and Nagoya University were among the institutions that experienced early outbreaks in the field of GCE. These institutions began to lead research in this area before 2017, and their influence has persisted to the present day. Conversely, Islamic Azad University, Lanzhou University, Tongji University, the Chinese Academy of Sciences, and the Medical School of Isfahan University are institutions that have recently emerged as significant contributors. The rise of these institutions may reflect the latest advancements in the field of GCE. It is noteworthy that, although the Chinese Academy of Sciences has experienced a surge in publications in recent years, it has consistently maintained a strong influence in this field.

To further understand the cooperation network among institutions, we conducted a cluster analysis using VOSviewer (Figure 4b). For this analysis, we set the minimum number of documents required for an institution at 20. Ultimately, 87 institutions met this criterion and were grouped into three clusters, represented by red (*n* = 42), green (*n* = 33), and blue (*n* = 10). The red cluster is centered around Shanghai Jiao Tong University, Nanjing University, Zhejiang University, and Sun Yat-sen University, forming the largest cluster. Most of these institutions are from China. The green cluster is centered on the National Cancer Center Japan, The University of Tokyo, the National University of Singapore, and Seoul National University, forming the second-largest cluster. Interestingly, these institutions are from different countries and do not include any from China.

Journals

A total of 4,655 documents were published across 190 journals. The top 20 journals in terms of the number of published articles are listed in Table 3. We found that the majority of these articles were published in journals within the Q1 and Q2 quartiles, with an average impact factor of 5.173. This indicates that research on GCE is of relatively high quality and can objectively reflect the role and mechanisms of GCE. Among the top 20 journals, 19 are categorized under the discipline of oncology. This suggests that research on GCE is primarily published in tumor-related journals, with relatively fewer articles appearing in specialized epigenetics-focused journals.

**Table 3:** The top 20 published journals.

Rank	Journal	Counts	JCR	IF
1	Oncotarget	110	/	5.16
2	World Journal of Gastroenterology	105	Q2	4.3
3	International Journal of Cancer	91	Q1	5.7
4	Oncogene	91	Q1	6.9
5	Frontiers in Oncology	86	Q2	3.5
6	Oncology Reports	86	Q2	3.8
7	Plos One	85	Q1	2.9
8	Cancers	71	Q2	4.5
9	Cancer Science	68	Q1	4.5
10	Oncology Letters	65	Q3	2.5
11	International Journal of Molecular Sciences	64	Q1	4.9
12	Cancer Research	63	Q1	12.5
13	Cancer Letters	59	Q1	9.1
14	International Journal of Oncology	59	Q1	4.5
15	Clinical Cancer Research	56	Q1	10.4
16	Scientific Reports	56	Q1	3.8
17	Gastric Cancer	54	Q1	6.0
18	Carcinogenesis	51	Q2	3.3
19	Tumor Biology	51	/	3.6
20	Anticancer Research	50	Q4	1.6

Author

We analyzed a total of 4,655 publications and identified the contributions of 19,229 authors. Table 4 lists the top 20 authors and their affiliated institutions. Among these authors, Ushijima Toshikazu from Japan has the highest number of published articles (*n* = 48), followed by the Chinese author Yu Jun (*n* = 35) and the Japanese author Shibata Tomoyuki (*n* = 24). Of the top 20 authors, 13 are from Japan, with most of these authors affiliated with Fujita Medical University. Although Fujita Medical University did not rank among the top 20 institutions, this indicates that the institution has a capable research team. This team, led by Shibata Tomoyuki, Tahara Tomomitsu, and Arisawa Tomiyasu, has considerable influence in the field of GCE. Chinese institutions have a significant advantage in the ranking. However, among the top 20 authors, only 3 are from China. This suggests that while there are many researchers in China, individual contributions are not as prominent. Furthermore, the affiliations and countries of the authors Kang Gyeong Hoon (Seoul National University, South Korea), Tan Patrick (National University of Singapore, Singapore), and Herman James G. (Johns Hopkins University, USA) are all within the top 20. This indicates that these authors are experts in the field of GCE research in their respective countries, and their institutions are also leading research centers in this field.

**Table 4:** The top 20 authors and affiliated institutions.

Rank	Authors	Institution	Country	Documents
1	Ushijima, Toshikazu	National Cancer Center Japan	Japan	48
2	Yu, Jun	Chinese University of Hong Kong	China	35
3	Shibata, Tomoyuki	Fujita Health University	Japan	24
4	Suzuki, Hiromu	Sapporo Medical University	Japan	22
5	Tahara, Tomomitsu	Fujita Health University	Japan	18
6	Arisawa, Tomiyasu	Fujita Health University	Japan	17
7	Kang, Gyeong Hoon	Seoul National University	South Korea	16
8	Guo, Mingzhou	Chinese People's Liberation Army General Hospital	China	15
9	Okubo, Masaaki	Fujita Health University	Japan	14
10	Hirata, Ichiro	Fujita Health University	Japan	14
11	Shinomura, Yasuhisa	Sapporo Medical University	Japan	14
12	Herman, James G	Johns Hopkins University	USA	14
13	Toyota, Minoru	Sapporo Medical University	Japan	13
14	Tan, Patrick	National University of Singapore	Singapore	13
15	Nagasaka, Mitsuo	Fujita Health University	Japan	12
16	Yuan, Yuan	China Medical University	China	11
17	Kim, Yong Sung	Korea Institute of Life Sciences and Biotechnology	South Korea	10
18	Kaneda, Atsushi	University of Tokyo	Japan	10
19	Nakamura, Masakatsu	Fujita Health University	Japan	9
20	Ohmiya, Naoki	Fujita Health University	Japan	9

**Figure 5:**
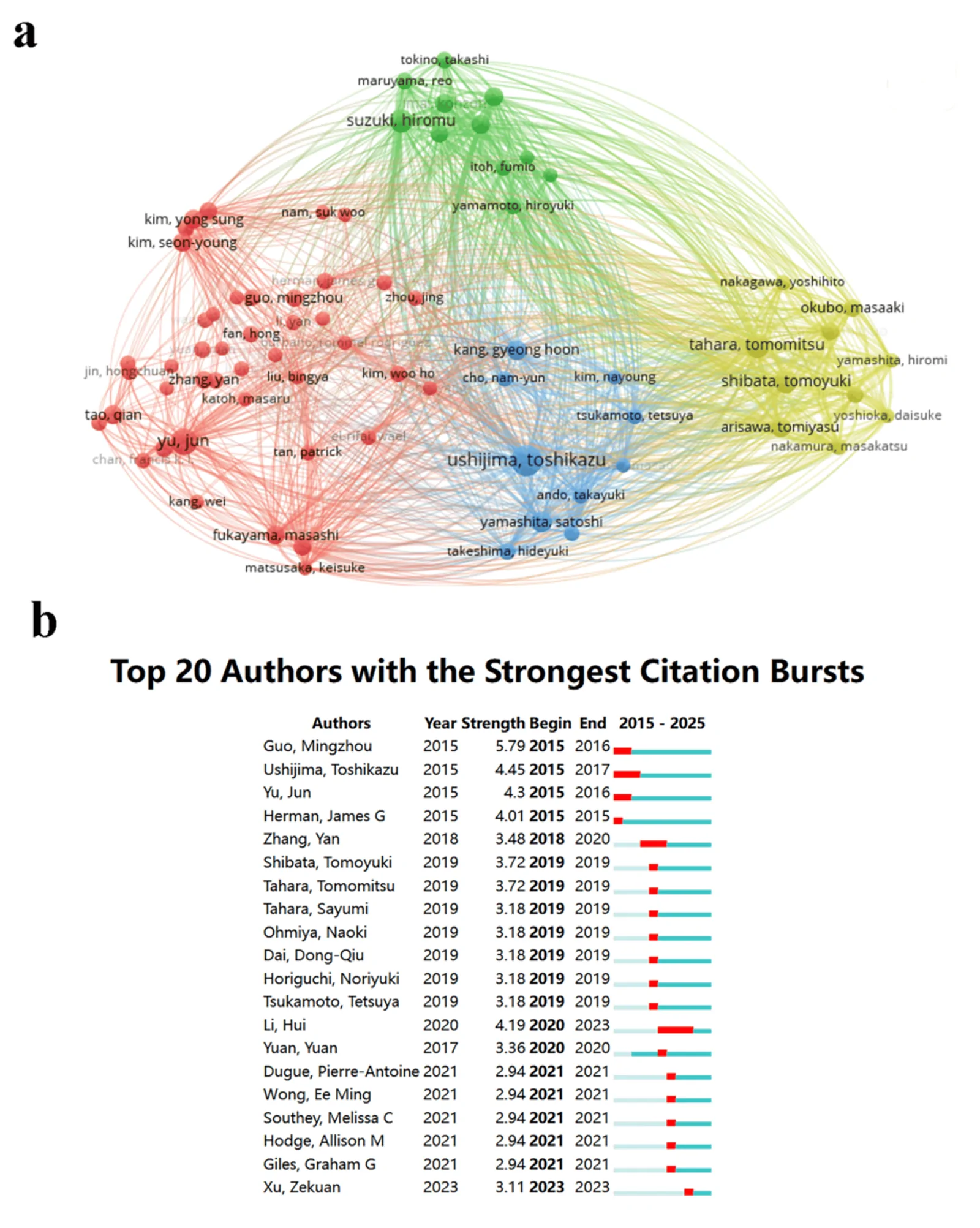
Visual analysis of authors in gastric cancer epigenetics (GCE) research. (a) Author cooperation clustering network diagram; (b) burst chart of the top 20 authors. Note: (a) The figure was drawn using VOSviewer software. The colors represent clusters, the node size represents the number of documents, and the lines represent the link strength. (b) The figure was drawn using CiteSpace software. The red bands represent the burst period, and the blue bands represent the buffer period.

To further investigate the collaborative relationships among authors, we set a minimum threshold of 15 published papers. Ultimately, 74 authors met this criterion. Through co-authorship coupling analysis, we identified four distinct clusters (Figure 5a), represented by red (*n* = 43), yellow (*n* = 10), blue (*n* = 10), and green (*n* = 11). The red cluster, centered around Yu Jun, Guo Mingzhou, and Kim Yong Sung, formed the largest cluster, with most authors from China. The blue cluster is centered on Ushijima Toshikazu, the yellow cluster on Shibata Tomoyuki, and the green cluster on Suzuki Hiromu. The core authors of these three clusters are all from Japan and are affiliated with different research institutions in Japan. Notably, the institutions associated with these authors are all ranked among the top 20 institutions. This suggests that these institutions are core centers for GCE research in Japan and play a significant leadership role in international cooperation. Surprisingly, none of the three smaller clusters included Chinese authors. This may indicate that, despite the large number of researchers in China, there is still room for improvement in international cooperation and exchanges.

Author burst analysis indicates that Guo Mingzhou, Ushijima Toshikazu, Yu Jun, and Herman James G were among the early authors to experience a surge in publication activity, and their influence has persisted to the present day. Yuan Yuan is identified as a representative author of the mid-term outbreak, while Xu Zekuan is noted as a representative of the recent outbreak (Figure 5b). Analyzing authors and their publications across different periods helps to reveal shifts in research hotspots within the field of GCE.

Keywords

Table 5 lists the top 20 keywords with the highest frequency of occurrence. Among these, the keywords related to epigenetics include "DNA methylation," "promoter methylation," and "hypermethylation," indicating that DNA methylation is the most extensively studied epigenetic mechanism in GCE. The keywords related to tumors include "colorectal cancer," "breast cancer," and "hepatocellular carcinoma," suggesting that these cancers may share common epigenetic mechanisms with GC or be regulated by similar mechanisms. Furthermore, "Helicobacter pylori" ranks ninth, highlighting its close association with the incidence of GC and its importance in GC research.

**Table 5:** The top 20 keywords and centrality.

NO.	Keyword	Frequency	Centrality
1	gastric cancer	2,666	0.2
2	dna methylation	1,302	0.08
3	expression	1,286	0.13
4	methylation	578	0.07
5	colorectal cancer	575	0.07
6	breast cancer	463	0.06
7	gene	457	0.03
8	carcinoma	449	0.07
9	helicobacter pylori	389	0.03
10	gene expression	385	0.06
11	cancer	358	0.04
12	tumor suppressor	355	0.04
13	hypermethylation	352	0.05
14	cells	332	0.04
15	metastasis	294	0.04
16	promoter methylation	289	0.04
17	proliferation	288	0.04
18	down regulation	274	0.03
19	hepatocellular carcinoma	268	0.07
20	Promoter hypermethylation	262	0.03

**Figure 6:**
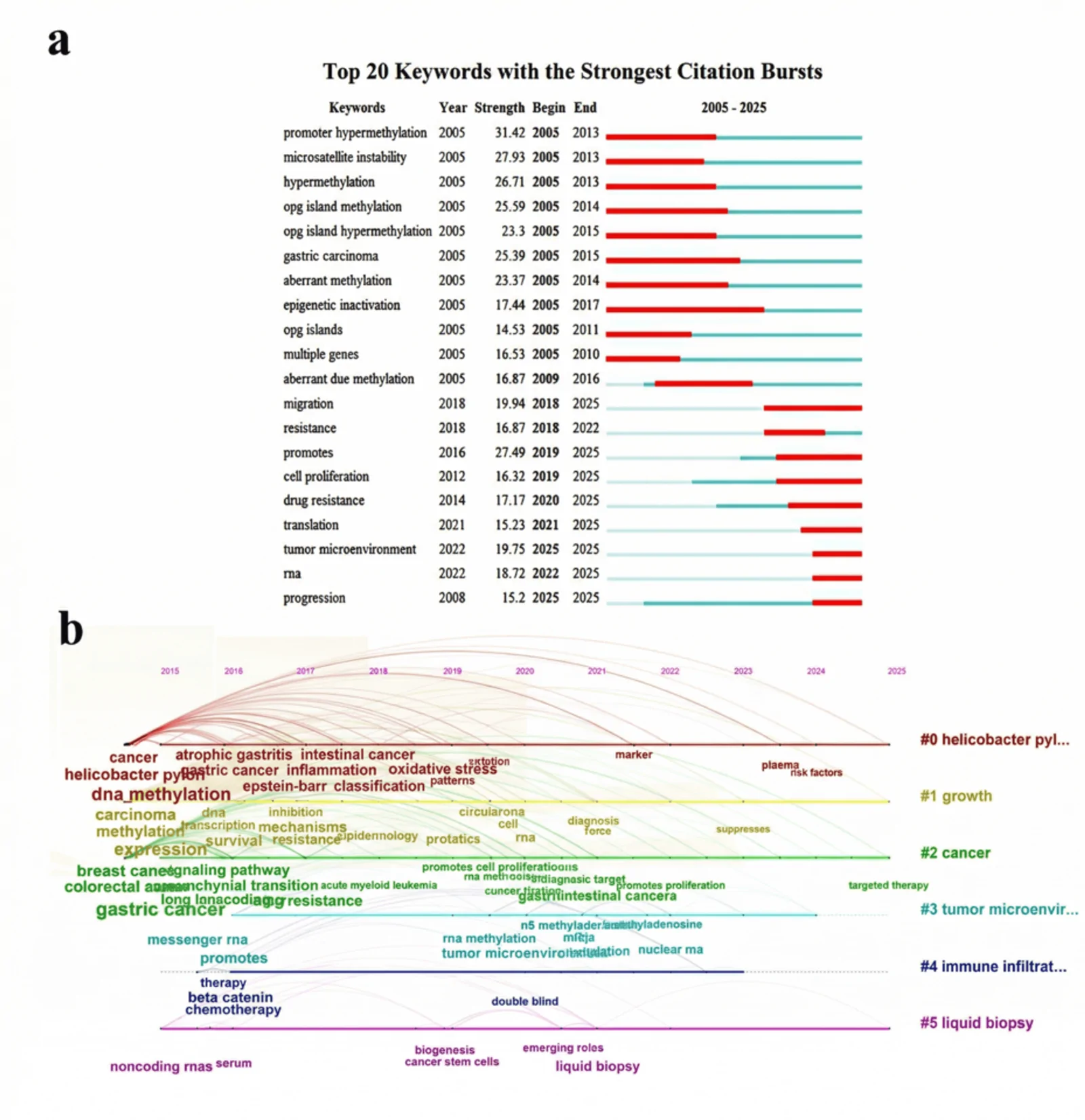
Visual analysis of keywords in gastric cancer epigenetics (GCE). (a) Burst chart of the top 20 keywords; (b) keyword timeline clustering graph. Note: (a) The figure was drawn using CiteSpace software. The red bands represent the burst period, and the blue bands represent the buffer period. (b) The figure was drawn using CiteSpace software. Colors represent clusters, node positions represent occurrence times, and lines represent link strengths.

To further understand the evolution trend of key words in GCE research, we conducted a burst analysis of the key words (Figure 6a). The results show that "promoter hypermethylation," "microsatellite instability," "hypermethylation," "CpG island methylation," and "aberrant methylation" were among the high-frequency keywords of the early outbreak. These keywords are all related to DNA methylation, indicating that DNA methylation has received attention in GC research relatively early. Among the keywords that have recently emerged, the popularity of "RNA" has persisted until now, indicating that the research focus on GCE has gradually shifted from DNA methylation to RNA-related mechanisms. Furthermore, "tumor microenvironment" is also a keyword that has emerged recently. In the research mechanism of GC, keywords have shifted from "migration," "resistance," "promotes cell," "proliferation," "drug resistance," and "translation" to "tumor microenvironment." This indicates that researchers' studies on tumors have shifted from individual characteristics to a comprehensive study involving multiple factors.

To further understand the recent shifts in research hotspots, we conducted a timeline clustering analysis of literature keywords over the past 10 years and identified a total of six clusters (Figure 6b). The epigenetics-related keywords, in chronological order, are: DNA methylation, mutation, ncRNA, lncRNA, mRNA, circulating RNA, and m6A. These keywords are primarily concentrated in cluster #3, which is associated with the tumor microenvironment. The tumor mechanism-related keywords, in sequence, are: signaling pathway, promotes cell proliferation, oxidative stress, cancer stem cells, and suppression. The tumor detection-related keywords are: liquid biopsy and marker. The tumor treatment-related keywords are: chemotherapy and targeted therapy. Based on the order in which these keywords appear, we can infer that the research focus on GC has shifted from specific molecular mechanisms to the overall tumor microenvironment, and treatment methods have gradually transitioned from traditional chemotherapy to targeted therapy.

## Discussion

This paper presents a systematic analysis of 4,655 publications in the field of GCE from a bibliometric perspective. Currently, research on GCE has reached its peak and entered a period of stable development. Against this backdrop, conducting a comprehensive analysis and summary of GCE research can help researchers understand the current global research status and provide inspiration for exploring new research hotspots and directions.

In the field of GCE, China has made the greatest contribution among all countries (*n* = 1,876, accounting for 40.30%). Additionally, the institution with the highest contribution (Nanjing University, *n* = 175) and the institution with the longest half-life (Chinese Academy of Sciences, half-life 7.5) are both from China. Among the top 20 institutions, 14 are from China. This indicates that Chinese researchers have a high enthusiasm for the epigenetic study of GC, which may also reflect the relatively high incidence of GC in China. The 2025 Global Cancer Statistics Report shows that China has the highest incidence of GC, followed by Japan and South Korea [1], all of which are Asian countries. Japan ranks third in the number of national literature publications in this field (*n* = 301, accounting for 6.47%). Among the top 20 institutions, only 2 are from Japan. However, among the top 20 authors, 13 are from Japan, and the majority of author collaboration clusters are centered around Japanese authors. This indicates that Japanese researchers have a strong influence in the field of GCE. The United States has the highest centrality (0.32) and ranks second in annual publication volume (*n* = 437, accounting for 9.39%), indicating that the United States places great emphasis on international cooperation and holds a core position in international collaboration. Johns Hopkins University in the United States (*n* = 79, ranked 12th) is a core research institution in the field of GCE in the United States.

The top three authors with the highest number of published articles are Ushijima T (*n* = 48, Japan), Yu Jun (*n* = 35, China), and Shibata T (*n* = 24, Japan). Ushijima T primarily investigates the mechanisms of DNA methylation in GC [8,9]. Yu Jun's team focuses on the prognostic predictive role of DNA methylation in GC [10,11]. Shibata T leads a capable research team at Fujita Medical University, which, centered around Shibata T, Tahara T, and Arisawa T, mainly studies the effects of gene polymorphisms on the methylation levels of gastric tissue and GC [12-14]. Herman JG (*n* = 14, ranked 12th) is a core author in the field of GCE research in the United States, with a primary research focus on DNA methylation changes and roles in various tumors, including GC [15,16]. Tan Patrick from the National University of Singapore (*n* = 13, ranked 14th) is a core researcher in this field in Singapore, with a broad research scope that includes chromatin remodeling [17], histone modification [18], RNA editing [19], miRNA [20], and DNA methylation [21]. Yuan Yuan (*n* = 11, ranked 16th, China) was a representative author during the mid-term outbreak, initially focusing on DNA methylation [22,23], before shifting to m6A modification [24,25].

The keyword "tumor microenvironment" emerged in 2022 and has remained popular to the present day. In the keyword timeline clustering analysis over the past nearly 10 years, this keyword has formed cluster #4, which includes a large number of terms related to epigenetics. This indicates that in the relationship between the tumor microenvironment and GC, epigenetic mechanisms are a key link connecting the two.

Among the top 20 keywords, five are related to DNA methylation, and among the early-emerging keywords, four are associated with DNA methylation. This indicates that DNA methylation research in GCE started earlier and is relatively mature. The term "hypermethylation" appears most frequently in keyword analyses, suggesting that DNA methylation levels are generally high in GC tissues. Furthermore, "promoter hypermethylation" appears in both keyword rankings and burst analyses, indicating that DNA hypermethylation in GC primarily occurs on CpG islands in the promoter regions.

Appendices A-E present the currently identified DNA methylation changes in GC and their roles in tumor development. Research by Zhou et al. shows that DNA methylation status in gastric tissue increases gradually in an age-dependent manner [26]. The research team also explored the relationship between accelerated methylation age (AA) in GC tissues and molecular subtypes, as well as patient survival. The epigenetic age acceleration (AA) of Epstein-Barr virus-related GC was 49.8 years (95% confidence interval (CI) 42.7-56.9), that of the microsatellite instability (MSI) subtype was 16.1 years (10.6-21.6), and that of the genomic stability (GS) subtype was 6.05 years (0.1-11.1). Studies have found that GC patients with accelerated tumor tissue aging have a better prognosis. Differentially methylated probes among patients with accelerated and slowed methylation aging were enriched in pathways related to bone morphogenetic protein (BMP) signaling, high mobility group box 1 (HMGB1) signaling, signal transducer and activator of transcription 3 (STAT3) signaling, and human embryonic stem cell pluripotency [27].

Research by Ando et al. also indicates that the methylation level of non-cancerous gastric mucosa in GC patients is 7.2-15.5 times higher than that in healthy volunteers [28], suggesting that the overall methylation level of gastric tissue is positively correlated with the risk of GC. The methylation level of specific genes can also reflect the pathological state of gastric tissue. For instance, among 4,654 GC patients and 3,669 non-malignant controls, MLH1 promoter methylation was significantly higher in GC samples than in gastric adenoma, chronic gastritis, adjacent tissues, normal gastric mucosa, and normal healthy blood samples, and MLH1 promoter methylation was age-related [29]. Clinically, a progressive increase in SPG20 methylation was observed in tissue samples ranging from gastritis (*n* = 34) to intestinal metaplasia (*n* = 33) and then to GC (*n* = 53) [30]. 

Although the relationship between DNA methylation status and the occurrence of GC has become a consensus, it still faces many challenges as a diagnostic marker. Tumor heterogeneity, individual differences, blood concentration, and the standardization of detection methods are the problems that need to be solved urgently [31,32]. The use of DNA methyltransferase (DNMT) inhibitors to reverse epigenetic silencing has been proven to enhance the cytotoxic activity of anti-cancer drugs and overcome drug resistance [33]. However, research by Park's team indicates that the concurrent use of the gene silencing reversal agent 5-Aza-CdR and FK228 in combination with oxaliplatin before or after GC cell therapy can produce antagonistic effects [34]. This suggests that the use of multiple gene silencers in combination with chemotherapy drugs requires more clinical data support. An in-depth exploration of the antagonistic mechanisms of combined medications is of great significance for the clinical application of gene silencing reversal agents and the response to drug resistance in GC.

The keyword outbreak analysis indicates that "RNA" is a keyword that experienced a mid-term outbreak, and non-coding RNA occupies a central position in the timeline clustering over the past 10 years. From this, we speculate that the epigenetic research of GC has evolved from DNA methylation and histone modification to non-coding RNA and, more recently, to m6A modification. Appendices F to H, respectively, present the current research status of non-coding RNA, histone modification, and m6A modification in GC. Studies have shown that histone modification and non-coding RNA often work in synergy with DNA methylation to jointly promote the progression of GC [35]. Currently, m6A modification has not yet formed a keyword outbreak, indicating that there is still substantial room for exploration of m6A modification in GC research. M6A modification is involved in various biological processes, including RNA splicing, maturation, translation, stability, and degradation [36]. In-depth exploration of the mechanisms of m6A modification in GC will contribute to the further development of GCE.

Microbial keywords related to the incidence of GC include "Helicobacter pylori" and "Epstein-Barr virus." Among these, "Helicobacter pylori" has a relatively high frequency of occurrence and has formed stable clusters, indicating that Helicobacter pylori infection is closely related to the pathogenesis of GC, and the related research is relatively mature. Appendices I and J, respectively, present the epigenetic changes in GC caused by Helicobacter pylori and Epstein-Barr virus. Interestingly, research on Helicobacter pylori is mainly concentrated in China, Japan, and South Korea. This may be related to susceptibility caused by racial genetic polymorphisms or external environmental factors. For instance, a SNP genotyping study of three histone modification genes conducted in South Korea on 1,100 GC patients and 1,249 healthy controls showed that all three genes were associated with GC susceptibility. Specifically, the genotypes of SNPs rs595227 and rs14497471 of the KDM6A gene, SNPs rs78633955 and rs11657063 of the KDM6B gene, and SNPs rs67648693 and rs1061037 of the EZH2 gene were significantly expressed. Furthermore, the probability of co-occurrence of these three susceptibility genes is also very high [37]. Although the incidence of GC after the cure of Helicobacter pylori infection is still higher than that of the uninfected [38], studies have shown that among healthy volunteers, the methylation level of individuals carrying Helicobacter pylori is 7.8-13.1 times higher than that of the uninfected. Additionally, there are differences in methylation levels between individuals who were previously infected with Helicobacter pylori and those currently infected, especially in protein-coding genes [28]. This suggests that Helicobacter pylori infection may accelerate the methylation age of certain genes in gastric tissue, thereby increasing the risk of GC even after cure. Therefore, epigenetic reversal after the cure of Helicobacter pylori may have a preventive effect on Helicobacter pylori-dependent GC, but this depends on the further development of GCE.

In the keyword timeline clustering analysis over the past nearly 10 years, "Immune infiltration" has formed cluster #5 but has not yet emerged as an explosive keyword. This indicates that "Immune infiltration" is a newly emerged research hotspot in recent years and currently holds great research potential. "Immune infiltration" often occurs concurrently with "Tumor microenvironment." The infiltration state of immune cells in tumors can alter the tumor microenvironment, which in turn affects patient prognosis by interfering with tumor cell immune escape. Epigenetic mechanisms play a significant role in the tumor immune microenvironment. Studies have shown that the methylation status of certain gene CpG sites can serve as an indicator of cancer immune infiltration and can be used to predict patients' responses to immunotherapy drugs [39]. For example, in GC tissues, the hypomethylation of the SYNC gene is associated with its upregulated expression, and the overexpression of the SYNC gene can promote the polarization of macrophages to the M2 type in the tumor immune microenvironment [40]. Furthermore, studies have shown that the overexpression of the reader protein YTHDF1 in GC tissues is significantly negatively correlated with the levels of CD8+ T cells, B cells, macrophages, dendritic cells (DC), and neutrophils in cancer tissues [41]. Tumor cell immune escape promotes tumor progression, and immune checkpoint proteins (such as PD-1, PD-L1, CTLA-4) play a key role in this process. Inhibitors targeting these immune checkpoints (such as anti-PD-1, anti-PD-L1, and anti-CTLA-4 monoclonal antibodies) can enhance the anti-tumor immune response of T cells but are mainly effective for "hot tumors." Epigenetic mechanisms can enhance the immune activity of "cold tumors" by influencing the tumor microenvironment. Studies have shown that histone acetylation mediated by ARID1A is positively correlated with CD8+ T cell infiltration and clinical prognosis. The application of deacetylase inhibitors effectively reversed the effect of ARID1A depletion on tumor progression and significantly enhanced the efficacy of immunotherapy [42]. Deletion of YTHDF1 can mediate the overexpression of interferon (IFN)-γ receptor 1, thereby stimulating a strong cytotoxic T lymphocyte response. Effective downregulation of YTHDF1 expression can inhibit the progression and metastasis of GC [43]. Although epigenetic mechanisms play a role in regulating tumor immune infiltration, they still face challenges due to poor tumor targeting and high systemic toxicity. You et al. developed an engineered small extracellular vesicle (sEV) with high CD47 expression and cycloarginine-glycine-aspartic acid (c(RGDyC)) modification for the effective delivery of YTHDF1 short-interfering RNA for the treatment of GC through epigenetics and immunomodulation [43]. The CD47 expression on engineered sEVs can competitively bind to signal regulatory protein α, thereby enhancing the phagocytic effect of tumor-associated macrophages on tumor cells. This effective and low-toxicity sEV shows great potential in inhibiting epigenetic regulatory factors, but more clinical data are still needed to verify its role in promoting immunotherapy.

Our study is the first to analyze the current research status and evolution of hotspots in the field of epigenetics related to GC from the perspective of bibliometrics. However, there are several limitations to this study. First, the choice of keywords may not fully capture all relevant research topics, as some emerging areas may have been overlooked. Second, the time span of the data collection, limited to publications from 2005 to 2025, excludes earlier important studies that could provide additional context. Third, the regional focus of the included studies, primarily from databases like Web of Science, may not fully represent global research trends, particularly from non-English speaking countries. Additionally, the limitations in software functionality, such as CiteSpace and VOSviewer, may affect the comprehensiveness of the network and clustering analysis, as certain complex relationships may not be fully captured. Finally, the use of only a single database may have resulted in the exclusion of relevant papers not indexed in Web of Science. Despite these limitations, this study provides a comprehensive and systematic overview of the field by integrating large-scale data and multi-dimensional analysis. We aim to address these issues in future research by expanding the scope of keyword selection, broadening the geographic coverage, and utilizing multiple databases and advanced software tools.

## Conclusions

This paper presents a systematic analysis of 4,655 epigenetic publications on GC from the past 20 years, using bibliometric methods. China has contributed the most publications, while the United States serves as the central country for cooperation in GCE. Japanese researchers wield considerable influence in this field. China, Japan, and South Korea have the highest incidence rates of GC, which may be related to genetic polymorphisms and epigenetic differences. Epigenetic modifications are crucial in tumorigenesis and changes in the tumor microenvironment. Among these, research on DNA methylation in GC is the most advanced. Its levels are positively correlated with GC incidence and hold significant potential for GC diagnosis. DNA methylation inhibitors, when combined with chemotherapy, play a vital role in overcoming chemotherapy resistance, though substantial clinical validation is still required. Current research hotspots have shifted from DNA methylation, histone modification, and non-coding RNA to m6A modification, which still offers broad research opportunities in GC. Helicobacter pylori and Epstein-Barr virus are two microorganisms associated with GC. Helicobacter pylori infection accelerates gastric tissue methylation age, alters epigenetic conformation, and persists even after infection clearance. Reversal of epigenetic age is anticipated to become an effective target for preventing Helicobacter pylori-associated GC. Immune infiltration is an emerging hotspot in GC immunotherapy. Epigenetic regulatory factors hold great potential for modulating the tumor immune microenvironment and are expected to advance GC immunotherapy further.

## Appendices

Appendix A

**Table 6:** Part 1: Regulation of DNA Methylation in Gastric Cancer.

Gene	Methylation status	Methylation position	Mechanism	DOI
hTERT	up	promoter	hTERT hypermethylation is related to telomerase expression	DOI10.3109/13547500903225912
SMARCA5	up	promoter	Promote the proliferation of tumor cells	DOI10.3109/07357907.2010.543365
SLFN11	up	promoter	Inhibit the growth of gastric cancer and enhance the ability of cisplatin-induced S-phase arrest and apoptosis of gastric cancer cells	DOI10.7150/jca.32511
RPRM	up	promoter	Generally hypermethylated in gastric cancer tissues	DOI10.1371/journal.pone.0168635
SEP9	up	/	The methylation of SEP9 increases and its expression is downregulated, promoting the growth, metastasis and spread of gastric cancer cells	DOI10.1155/2022/3433406
ECRG4	up	promoter	The hypermethylation of ECRG4 downregulates its expression and increases its invasiveness	DOI10.2147/OTT.S161200
rpL40	up	/	SMYD5 mediates the methylation of rpL40 to promote the metastasis of gastric cancer	/
Somatostatin (SST)	up	promoter	Hypermethylation of SST inhibits the expression of SST and promotes the development of gastric cancer	DOI10.1007/s10620-010-1422-z
RELN	up	promoter	Hypermethylation of RELN inhibits the expression of RELN and promotes the development of gastric cancer	DOI10.3892/ijo_00000478
PIP4P2	/	/	High expression of PIP4P2 activates PI3K/AKT signal transduction and induces tumor progression	DOI10.1016/j.labinv.2023.100170
eEF1A2	down	/	Abnormal hypomethylation and high mRNA expression of eEF1A2 play a role in gastric cancer	DOI10.1007/s00428-024-03765-0
ERas	down	promoter	The absence of ERas methylation increases the expression of ERas and promotes the re-expression of embryonic oncogenes in gastric cancer	DOI10.3892/ijo_00000414
DLL1	down	/	DLL1 demethylation promotes the expression of DLL1 and activates the Notch signaling pathway to drive the occurrence of gastric cancer	/
CDH1	up	exon	Hypermethylation of CDH1 inhibits the editing of CDH1 and promotes the occurrence of gastric cancer	DOI10.1186/s12885-015-1983-5

Appendix B

**Table 7:** Part 2: Regulation of DNA Methylation in Gastric Cancer (Continued).

Gene	Methylation status	Methylation position	Mechanism	DOI
HOXA11	up	promoter	Hypermethylation silencing of HOXA11 promotes the proliferation, migration and invasion of GC by activating the Wnt signaling pathway	DOI10.2217/EPI.14.92
COX-2	up	promoter	COX-2 hypermethylation silencing improves the prognosis of patients with gastric cancer	DOI10.1200/JCO.2006.09.8921
PKD1	up	promoter	Hypermethylation silencing of PKD1 promotes the occurrence of gastric cancer	DOI10.1093/carcin/bgm291
DFNA5	up	5' -UTR	Hypermethylation silencing of DFNA5 promotes the occurrence of gastric cancer	DOI10.1111/j.1349-7006.2006.00351.x
CHFR	up	5' -UTR	Hypermethylation silencing of CHFR promotes the occurrence of gastric cancer by affecting the function of mitotic checkpoints	/
CCDC67	up	promoter	Hypermethylation silencing of CCDC67 promotes the occurrence of gastric cancer	DOI10.1093/carcin/bgs178
FGFR2	down	promoter	The methylation status of FGFR2 is downregulated in the blood of patients with gastric cancer and can be used as a biomarker	DOI10.1007/s11033-023-09082-0
TUSC1	up	/	Hypermethylation silencing of TUSC1 promotes the metastasis of gastric cancer	DOI10.1002/jso.23614
GDF1	up	promoter	Epigenetic silencing of GDF1 disrupts SMAD signals and enhances the development of gastric cancer	DOI10.1038/onc.2015.276
HAI-2/ SPINT2	up	promoter	Epigenetic inactivation of HAI-2/SPINT2 promotes the occurrence of gastric cancer	DOI10.1002/ijc.25161
PRDM5	up	5' -UTR	Hypermethylation silencing of PRDM5 promotes the occurrence of gastric cancer	DOI10.1158/1078-0432.CCR-07-0305
HINT1	up	promoter	Hypermethylation silencing of HINT1 promotes the occurrence of gastric cancer	DOI10.3892/ijo.2011.994
GATA2	up	/	GATA2 methylation silencing promotes the progression of gastric cancer by promoting the expression of GATA6	DOI10.1038/onc.2017.397
E-cadherin	down	promoter	Methionine deficiency reduces the promoter methylation of E-cadherin, promotes its expression, and reduces cell adhesion and migration	DOI10.1007/s11605-012-1996-1
CDH1	up	enhanser	Hypermethylation of CDH1 affects the expression of E-cadherin and is involved in the progression of gastric cancer	DOI10.3390/ph14050457

Appendix C

**Table 8:** Part 3: Regulation of DNA Methylation in Gastric Cancer (Continued).

Gene	Methylation status	Methylation position	Mechanism	DOI
HRK	up	/	HINT1 hypermethylation silencing inhibits doxorubicin-induced apoptosis in gastric cancer	/
DUSP9	up	promoter	Epigenetic silencing of DUSP9 induces the proliferation of human gastric cancer by activating the JNK signal	DOI10.3892/or.2015.3998
LIMS2	up	5' -UTR	Epigenetic silencing of LIMS2 promotes the migration of gastric cancer cells	DOI10.1016/j.bbrc.2006.08.128
ATP2A3	up	promoter	Methylation silencing of ATP2A3 inhibits the expression of SERCA3 and reduces the apoptosis of gastric cancer cells	DOI10.1002/mc.22978
SFRP	up	/	SFRP methylation silencing promotes the proliferation of gastric cancer cells through the Wnt pathway	DOI10.1038/sj.onc.1210259
Dies1	up	promoter	The hypermethylation silencing of Dies1 reduces tumor growth	DOI10.1038/srep34860
DAPK	up	promoter	Hypermethylation silencing of DAPK promotes the progression of gastric cancer	DOI10.1097/CEJ.0b013e32834c9caa
DCBLD2	up	promoter	Hypermethylation silencing of DCBLD2 promotes the proliferation and invasion of gastric cancer cells	DOI10.1158/1541-7786.MCR-07-0142
MZB1	up	promoter	Hypermethylation silencing of MZB1 enhances the proliferation, invasion and migration abilities of gastric cancer cell lines	DOI10.1002/ijc.30286
IFITM1	down	promoter	Hypomethylation of IFITM1 increases its expression and promotes the migration and invasion of gastric cancer cells	DOI10.1016/j.ajpath.2012.03.027
BACH2	up	promoter	Hypermethylation silencing of BACH2 promotes the progression of intestinal-type gastric cancer	DOI10.1016/j.canlet.2014.05.009
CDH1	up	promoter	Hypermethylation and mutant inactivation of CDH1 occur frequently in hereditary diffuse gastric cancer (HDGC)	DOI10.1053/j.gastro.2009.02.065
GSN	up	promoter	Tumor-associated macrophage-mediated hypermethylation silencing of GSN promotes the progression of gastric cancer	DOI10.1158/2326-6066.CIR-16-0295
GATA6	up	promoter	Abnormal JAK/STAT signals are involved in the occurrence of gastric cancer by inducing methylation silencing of GATA6	DOI10.3390/ijms17091467
DLC-1	up	promoter	Hypermethylation silencing of DLC-1 is common in gastric cancer	DOI10.1038/sj.onc.1206573

Appendix D

**Table 9:** Part 4: Regulation of DNA Methylation in Gastric Cancer (Continued).

Gene	Methylation status	Methylation position	Mechanism	DOI
BCL6B	up	promoter	Hypermethylation silencing of BCL6B promotes the progression of gastric cancer	DOI10.1136/gutjnl-2011-300411
RASGRF1	up	/	RASGRF1 methylation is closely related to epigenetic field defects in the stomach, and it may be a useful biomarker for identifying individuals at high risk of gastric cancer	DOI10.1158/1940-6207.CAPR-12-0056
NR4A3	up	promoter	Abnormal JAK/STAT3 signals promote the progression of gastric cancer by silencing NR4A3 through hypermethylation	DOI10.1038/srep31690
IRX1	up	promoter	PRMT5 promotes the tumorigenicity and metastasis of gastric cancer cells by epigenetic silencing of IRX1	DOI10.1016/j.bbadis.2018.05.015
DACH1	up	promoter	Epigenetic silencing of DACH1 induces the invasion and metastasis of gastric cancer by activating TGF-β signal transduction	DOI10.1111/jcmm.12325
OLFM4	up	promoter	The down-regulation of OLFM4 enhances the invasion of gastric cancer cells by activating the FAK signal	DOI10.5483/BMBRep.2015.48.11.130
SFRP2	up	promoter	The down-regulation of SFRP2 promotes the progression of gastric cancer	DOI10.1038/sj.bjc.6603968
hSRBC	up	promoter	The down-regulation of hSRBC contributes to the malignant progression of gastric tumors	DOI10.1002/ijc.23166
LAMA3	up	promoter	LAMA3 methylation silencing promotes the progression of gastric cancer	DOI10.3892/ijo.2011.1048
ATP4B	up	/	Inhibition of ATP4B expression reduces the sensitivity of gastric cancer to docetaxel	DOI10.3727/096504016X14734735156265
DLEC1	up	promoter	Silencing of DLEC1 methylation promotes the progression of gastric cancer	/
SHP1	up	promoter	Down-regulation of SHP1 regulates multiple target genes through the JAK2/STAT3 pathway, promoting the proliferation, migration and invasion of gastric cancer cells	DOI10.1007/s13277-015-4228-y
PD-L1	up	/	PD-L1 DNA methylation is negatively correlated with the expression of PD-L1 in tumor samples	DOI10.1016/j.gene.2020.145376
SPG20	up	promoter	Methylation silencing of SPG20 promotes the progression of gastric cancer	DOI10.1371/journal.pone.0218338

Appendix E

**Table 10:** Part 5: Regulation of DNA Methylation in Gastric Cancer.

Gene	Methylation status	Methylation position	Mechanism	DOI
hMLH1	up	promoter	hMLH1 is involved in the development of multiple gastric cancers under the MSI pathway through promoter hypermethylation inactivation	DOI10.3164/jcbn.40.203
MDR1	up	/	MDR1 methylation inhibits the expression of MDR1 in gastric cancer and promotes tumor progression	DOI10.1186/1471-230X-8-33
PSD	up	/	Methylation of PSD inhibits the expression of PSD in gastric cancer and promotes tumor progression	DOI10.3892/ijo.2014.2736
RPRML	up	/	Methylation of RPRML inhibits the expression of RPRML in gastric cancer and promotes tumor progression	DOI10.3390/ijms21249472
PLCD1	up	/	Methylation of PLCD1 inhibits the expression of PLCD1 in gastric cancer and promotes tumor progression	DOI10.1038/onc.2009.92
PAX5	up	promoter	Methylation silencing of PAX5 promotes the migration and invasion of gastric cancer cells	DOI10.1038/onc.2011.511
CIITA	up	/	The down-regulation of CIITA methylation prompts gastric cancer cells to evade immune surveillance	DOI10.1038/sj.onc.1208144
MLH1	up	promoter	Methylation of the MLH1 promoter is significantly associated with microsatellite instability (MSI) gastric cancer	DOI10.1159/000486354
SYNC	down	/	SYNC increases the M2 polarization of macrophages in the immune microenvironment of gastric tumors and promotes gastric cancer infiltration	DOI10.1089/gtmb.2020.0131

Appendix F

**Table 11:** The regulation of non-coding RNA in gastric cancer.

Gene	Methylation status	Methylation position	Mechanism	DOI
miR137	up	promoter	Methylation mediates the down-regulation of miR137, thereby stimulating the expression of FMNL2 and promoting the occurrence of gastric cancer	/
lncRNA KCNK15-AS1	up	/	Methylation mediates the down-regulation of RNA KCNK15-AS1 and promotes the occurrence of gastric cancer through the KCNK15-AS1-DNMT1-MAPK and KCNK15-AS1-HDAC1-Akt pathways	DOI10.2147/CMAR.S186002
miR-196s	down	promoter	Hypomethylation of miR-196s promotes the expression of miR-196s and increases the risk of gastric cancer	DOI10.1002/gcc.20804
miR-34b	up	/	Hypermethylation silencing of miR-34b is associated with poor clinicopathological characteristics	DOI10.1002/ijc.25919
miR-129-3p	up	/	Hypermethylation silencing of miR-129-3p is associated with poor clinicopathological characteristics	DOI10.1002/ijc.25919
miR-1271	up	/	The epigenetic silencing of miR-1271 targeting MEK1 and TEAD4 is involved in the progression of gastric cancer	DOI10.1002/cam4.1605
miR-10b	up	promoter	Methylation silencing of miR-10b promotes the expression of MAPRE1 and is involved in the progression of gastric cancer	DOI10.4161/epi.6.6.15874
miR-129-2	up	/	The epigenetic silencing of miR-129-2 promotes the expression of SOX4 and participates in the progression of gastric cancer	DOI10.1016/j.bbrc.2010.03.121
mir -31	up	promoter	Methylation silencing of mir-31 negatively regulates the expression of HDAC2 and is involved in the progression of gastric cancer	DOI10.1155/2017/5348490
miR-338-3p	up	/	Epigenetic silencing of miR-338-3p promotes the proliferation and migration of gastric cancer cells	DOI10.1371/journal.pone.0066782
miR-200b	up	promoter	Cafs-induced methylation silencing of miR-200b promotes the invasion and migration of gastric cancer	DOI10.1093/carcin/bgu232
miR-34b/c	up	/	Methylation silencing of miR-34b/c promotes the progression of gastric cancer	DOI10.1093/carcin/bgq203
miRNA-9	up	promoter	miRNA-9 methylation silencing promotes the progression of gastric cancer	DOI10.3892/ijo.2014.2667
miR-193a	up	/	Transcriptional inhibition of miR-193a leads to overexpression of YWHAZ and promotes tumor progression	DOI10.3389/fonc.2021.575667

Appendix G

**Table 12:** The regulation of histone modification in gastric cancer.

Gene	Histone modification	Mechanism	DOI
GKN1	H3K9 Trimethylation	The downregulation of GKN1 is associated with high levels of H3K9 trimethylation and SUV39H1 recruitment of the GKN1 promoter	DOI10.18632/oncotarget.14817
RhoE	Histone acetylation	Histone deacetylation modification down-regulates the expression of RhoE and promotes the progression of gastric cancer	DOI10.3892/or_00001058
CDH1	Histone acetylation	Inhibit the editing of CDH1 and promote the occurrence of gastric cancer	DOI10.1186/s12885-015-1983-5
PRDM5	H3L27 Trimethylation	H3L27 Trimethylation inhibits the expression of PRDM5 and promotes the occurrence of gastric cancer	DOI10.1158/1078-0432.CCR-07-0305
BRD4	Histone acetylation	Promote the binding of BRD4 to the c-MYC promoter and participate in the progression of gastric cancer	DOI10.1002/jcb.26264
HRK	Histone acetylation	Inhibit the expression of HRK and reduce the sensitivity of doxorubicin in gastric cancer	/
DLC-1	H3 and H4 acetylation	Inhibit the expression of DLC-1 in gastric cancer	DOI10.1038/sj.onc.1206573
ATP4B	H3L9 deacetylation	Inhibition of ATP4B expression reduces the sensitivity of gastric cancer to docetaxel	DOI10.3727/096504016X14734735156265
MDR1	Histone deacetylation	Histone deacetylation inhibits the expression of MDR1 in gastric cancer and promotes tumor development	DOI10.1186/1471-230X-8-33
PCAT18	H3K27 Trimethylation	H3K27 methylation modification inhibits the expression of PCAT18 and promotes tumor progression	DOI10.7150/jca.63415

Appendix H

**Table 13:** Epigenetic regulation of m6A modification in gastric cancer.

Gene	Modification State	Modified Enzyme	Mechanism	DOI
SUV39H2	up	METTL3-IGF2BP2	M6A modification upregulates the expression of SUV39H2 to promote GC proliferation and inhibit cell apoptosis	/
IFN- γ1	/	YTHDF1	The deletion of YTHDF1 mediates the overexpression of interferon (IFN)- γ receptor 1, enhances the IFN- γ response, and stimulates the response of highly cytotoxic T lymphocytes	DOI10.1002/adma.202204910

Appendix I

**Table 14:** Epigenetic changes caused by Helicobacter pylori infection in gastric cancer.

Gene	Methylation status	Methylation position	Mechanism	DOI
TFF2	up	/	MUC1 reduces and upregulates the methylation of TFF2, lowers the expression of TFF2, and leads to a poor prognosis for patients	DOI10.1186/s13148-020-00832-6
Tensin 4 （TNS4）	down	promoter	TNS4 hypomethylation promotes TNS4 expression and increases the colony formation and cell migration of gastric cancer cells	DOI10.14348/molcells.2023.2148
HINT1	up	promoter	Hypermethylation silencing of HINT1 promotes the occurrence of gastric cancer	DOI10.3892/ijo.2011.994
miR-124a-3	up	/	The methylation level increases in individuals who have eradicated Helicobacter pylori and can be used as an independent marker	DOI10.1007/s10120-018-0803-4
miR-124	up	/	miR-124 methylation silencing promotes SMOX expression and mediates DNA damage	DOI10.1038/onc.2016.91
MUC17	up	promoter	The down-regulation of MUC17 promotes the transport of Helicobacter pylori CagA into gastric cancer cells and enhances the growth of gastric cancer cells	DOI10.1007/s10120-019-00932-0
TRPM3	up	promoter	The increase of methylation in the TRPM3 promoter inhibits the expression of microRNA-204 and promotes the progression of gastric cancer	DOI10.1093/carcin/bgz143

Appendix J

**Table 15:** Epigenetic changes caused by Epstein-Barr virus infection in gastric cancer.

Gene	Methylation status	Methylation position	Mechanism	DOI
HINT1	up	promoter	Hypermethylation silencing of HINT1 promotes the occurrence of gastric cancer	DOI10.3892/ijo.2011.994
IHH	up	/	Hypermethylation silencing of IHH affects the occurrence of gastric cancer	DOI10.1053/j.gastro.2014.08.036
TRABD	up	/	Hypermethylation silencing of TRABD affects the occurrence of gastric cancer	DOI10.1053/j.gastro.2014.08.036
