# Correction: Global Research Trends in Gastric Cancer Epigenetics From 2005 to 2025

**DOI:** 10.7759/cureus.c492

**Published:** 2026-10-14

**Authors:** Didi Yuan, Yanling Li, Hong Yi, Yan Lei, Yanhong Zhou

**Affiliations:** 1 Department of Blood Transfusion, The Affiliated Cancer Hospital of Xiangya School of Medicine Central South University, Hunan Cancer Hospital, Changsha, CHN; 2 Basic School of Medicine, Central South University, Central South University, Changsha, CHN; 3 Department of Nuclear Medicine, The Affiliated Cancer Hospital of Xiangya School of Medicine Central South University, Hunan Cancer Hospital, Changsha, CHN; 4 Research Center of Carcinogenesis and Targeted Therapy, Xiangya Hospital of Central South University, Changsha, CHN; 5 Cancer Research Institute, Basic School of Medicine, Central South University, Changsha, CHN

This article has been corrected to update affiliations to reflect the full names of The Affiliated Cancer Hospital of Xiangya School of Medicine, Central South University, Hunan Cancer Hospital, and Cancer Research Institute, Basic School of Medicine, Central South University. Dr. Hong Yi’s affiliation has been changed to Xiangya Hospital of Central South University.

